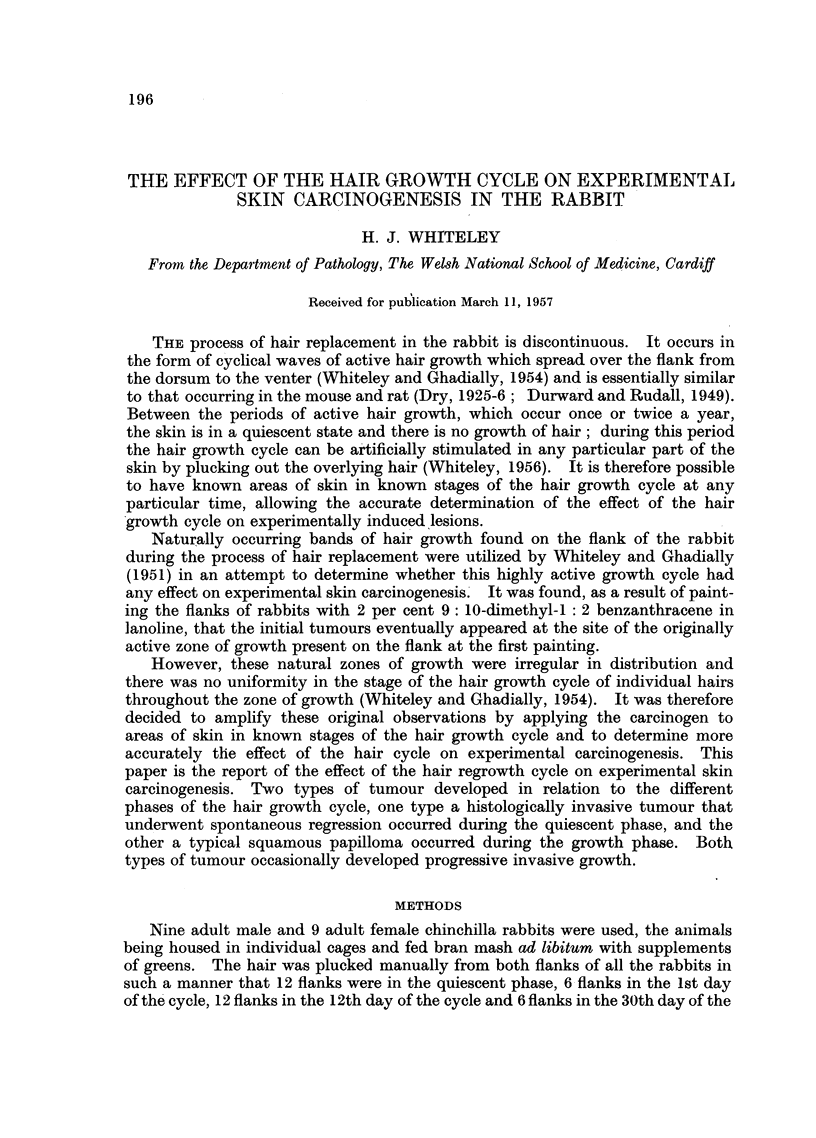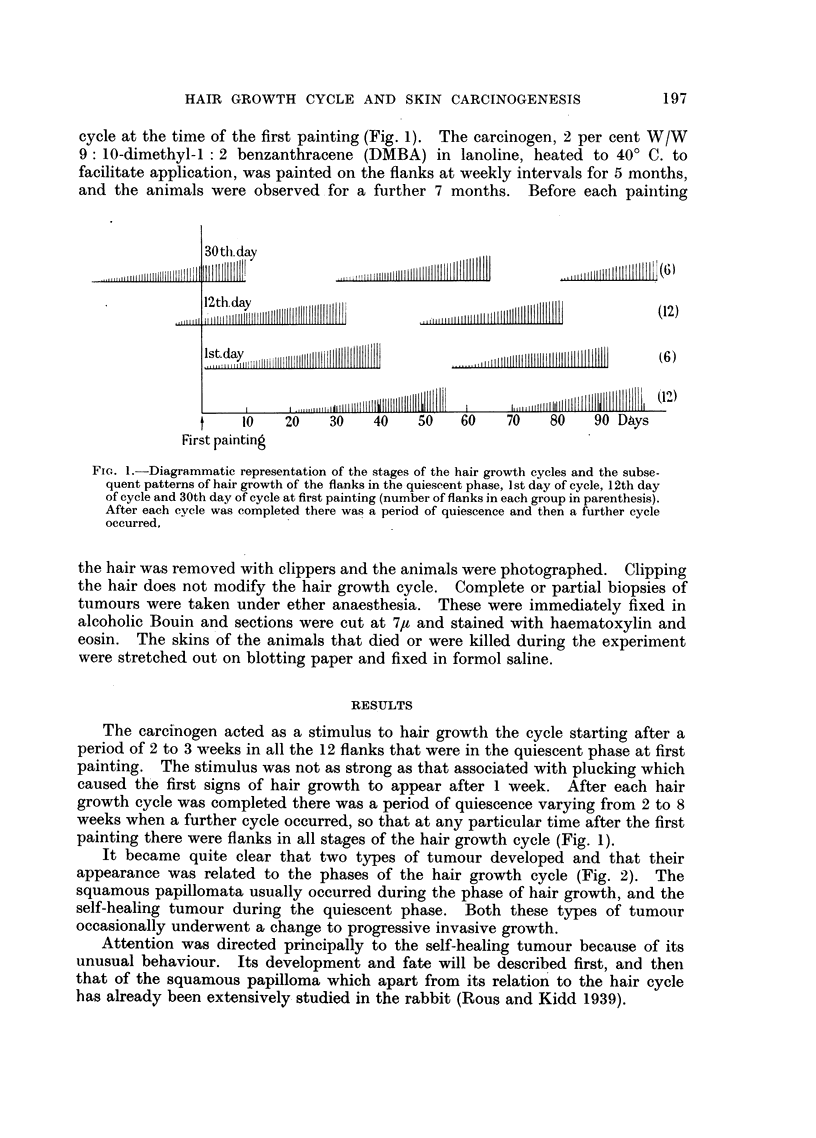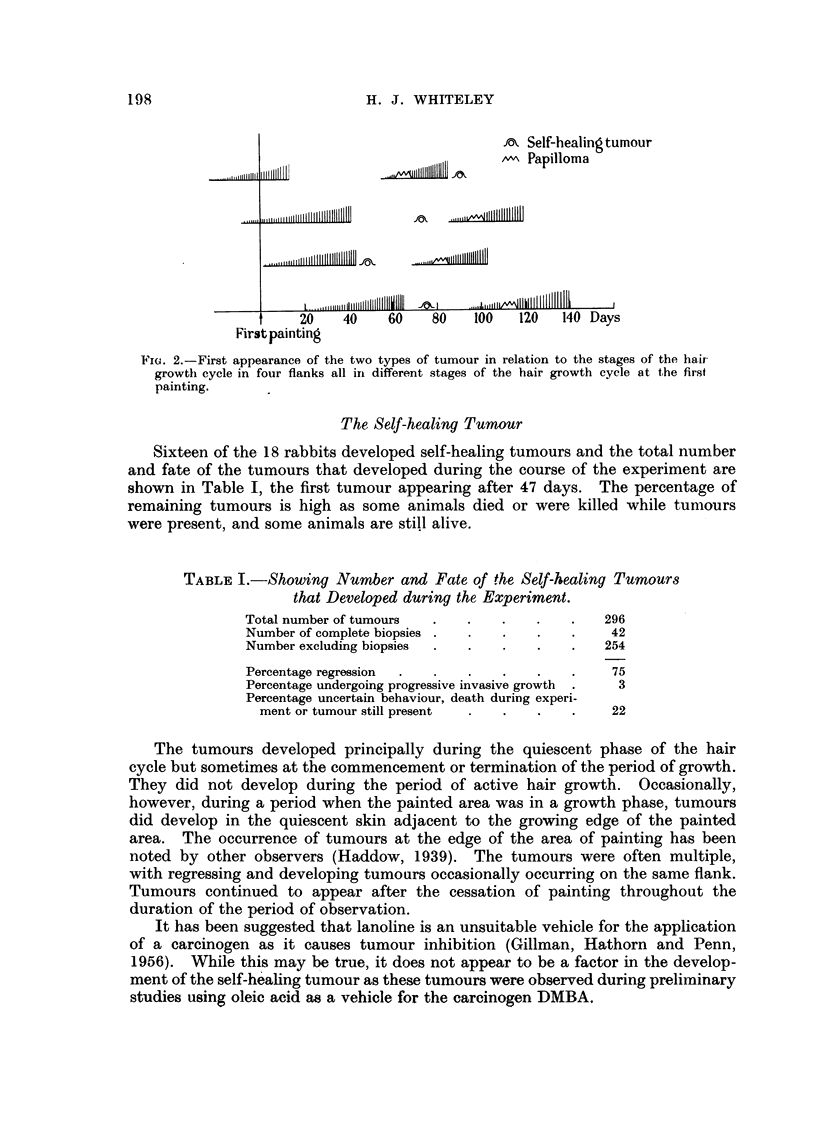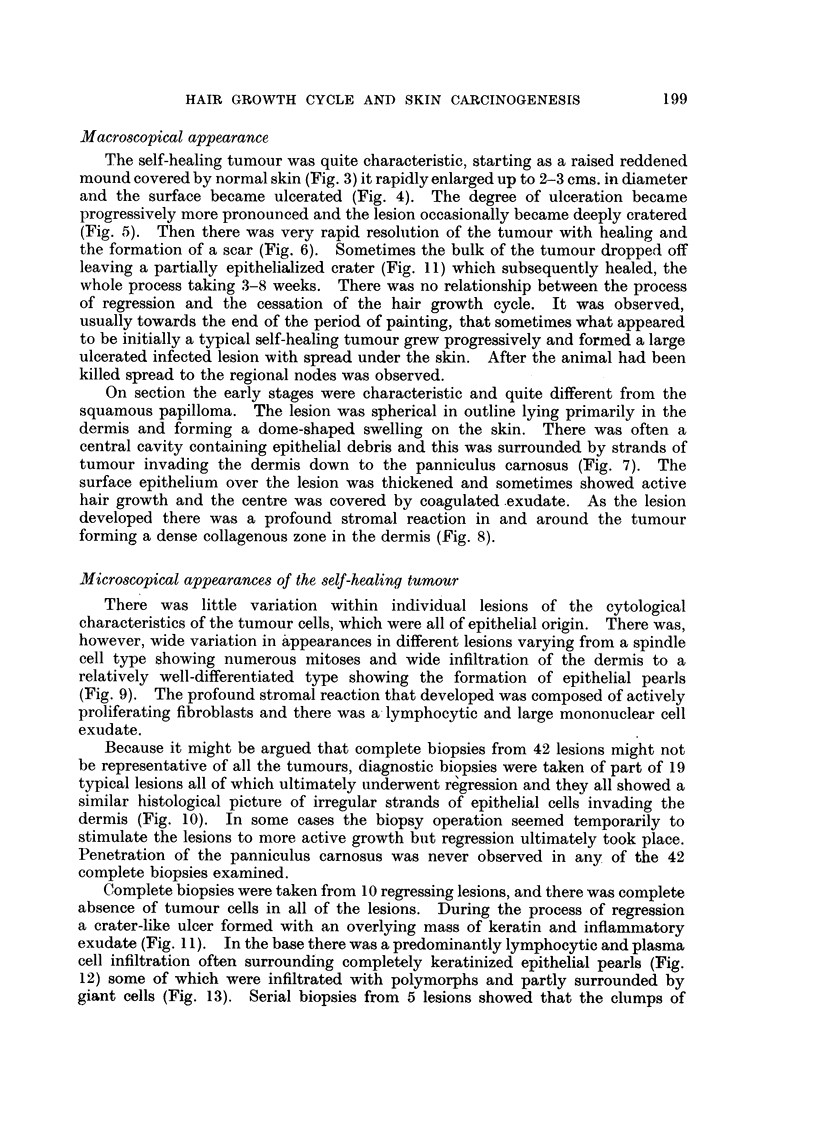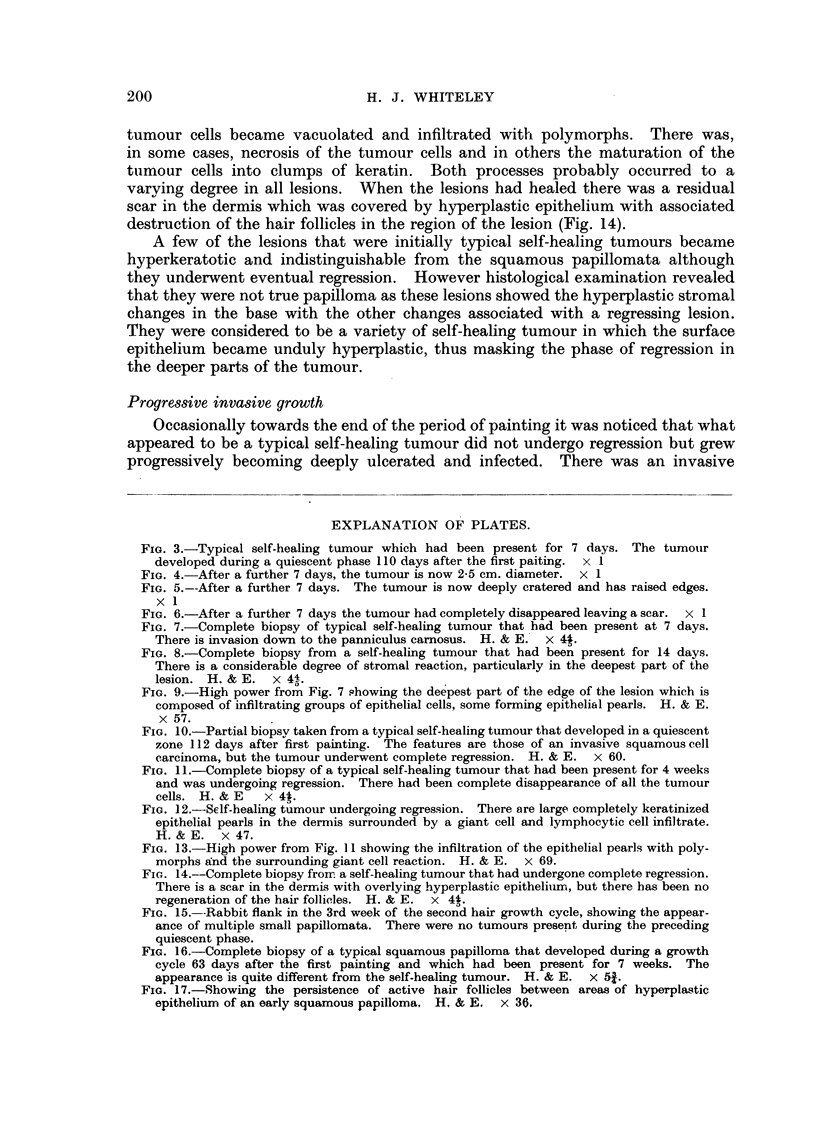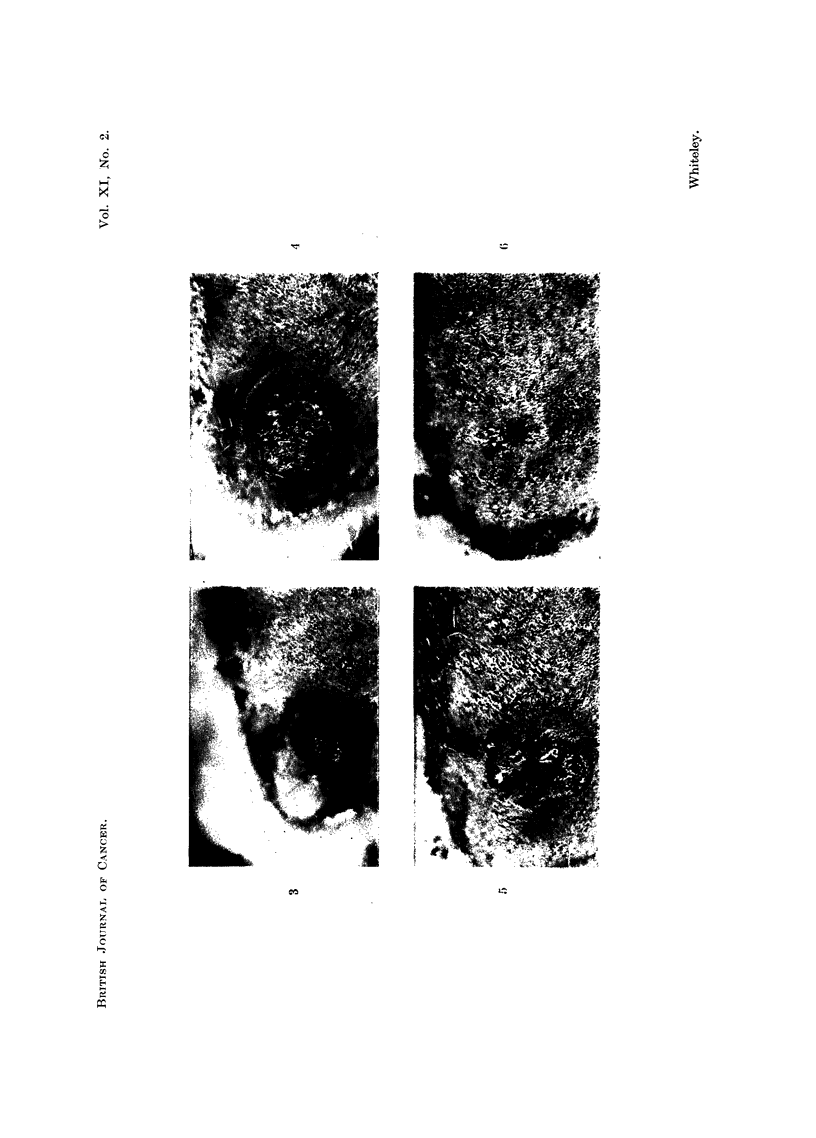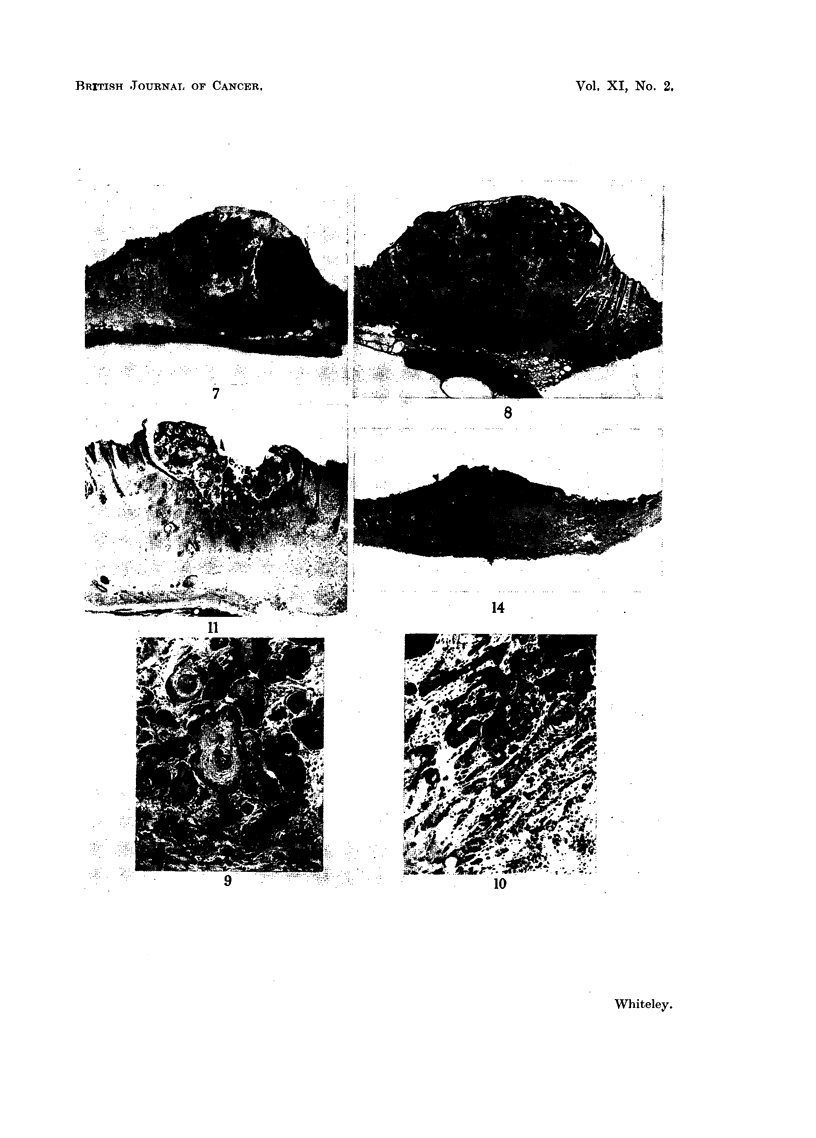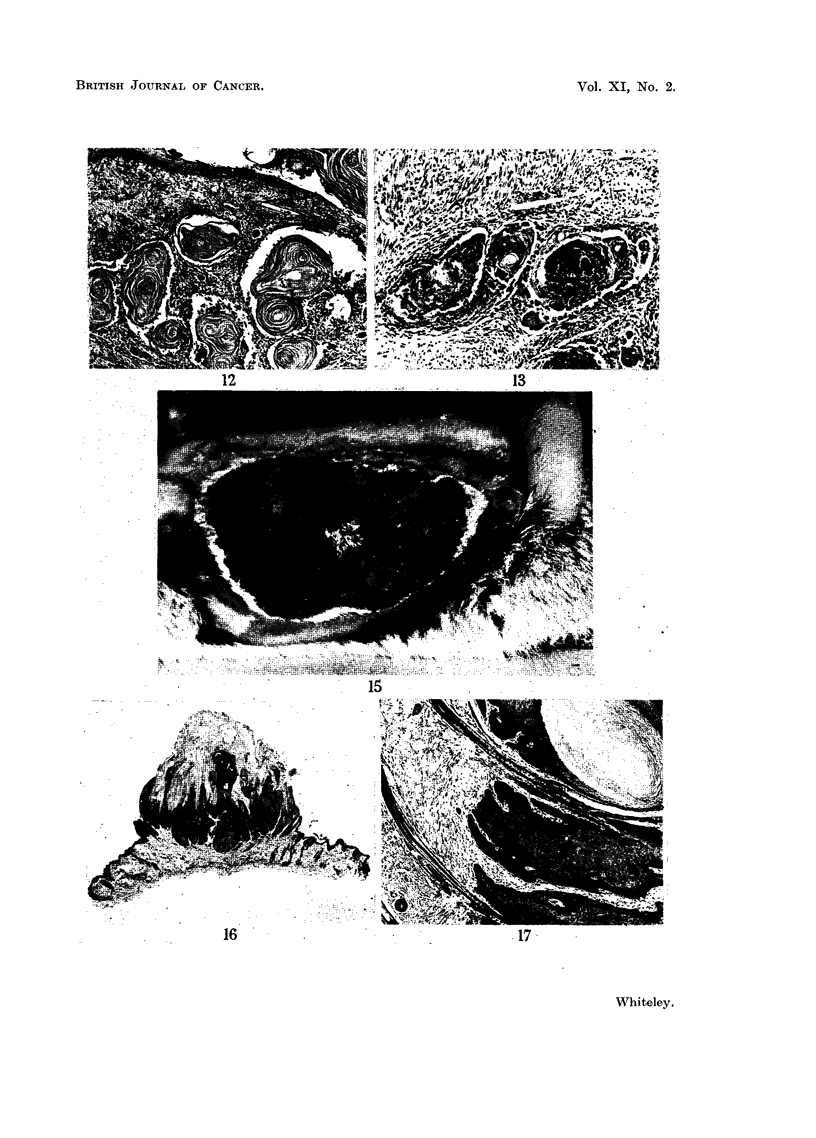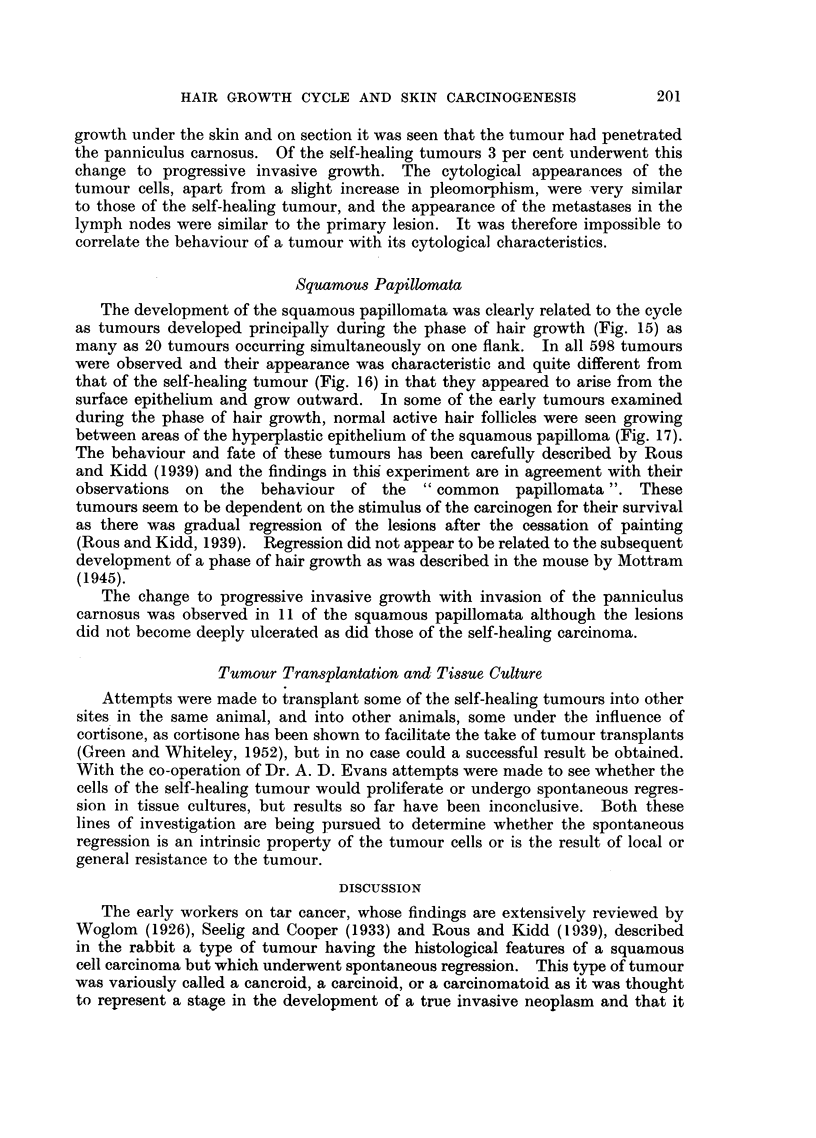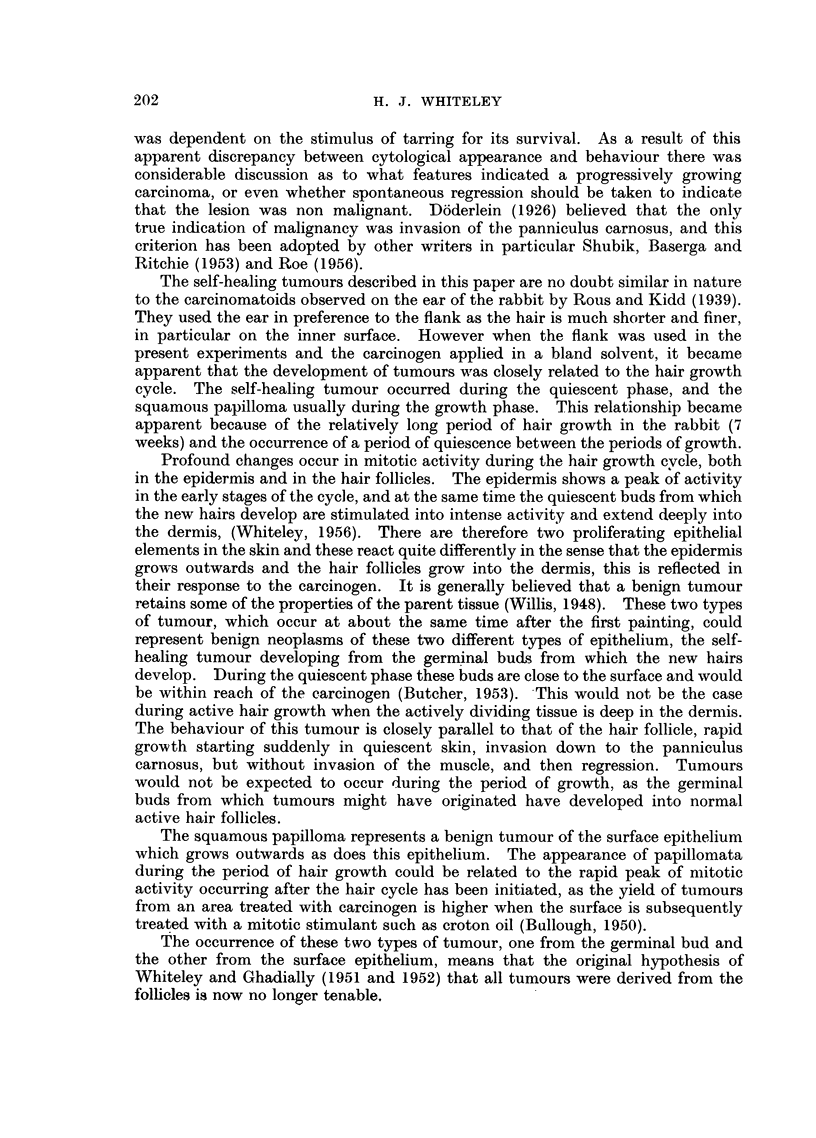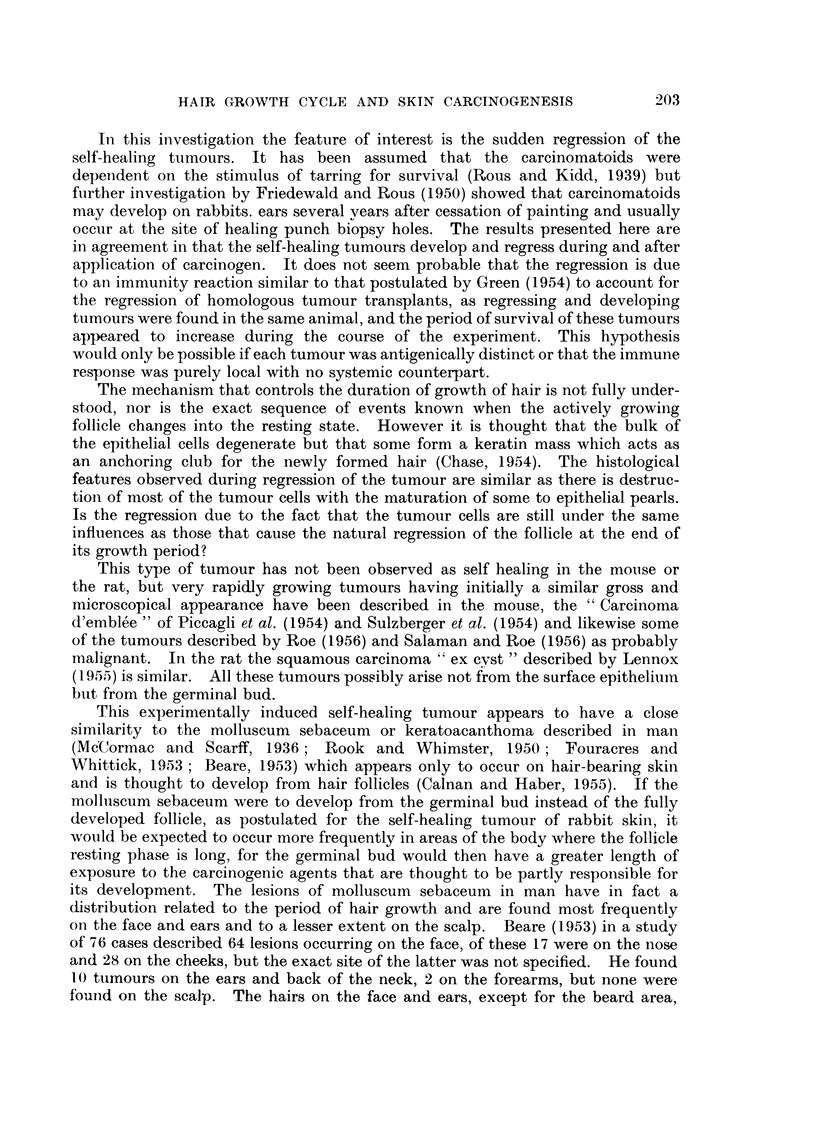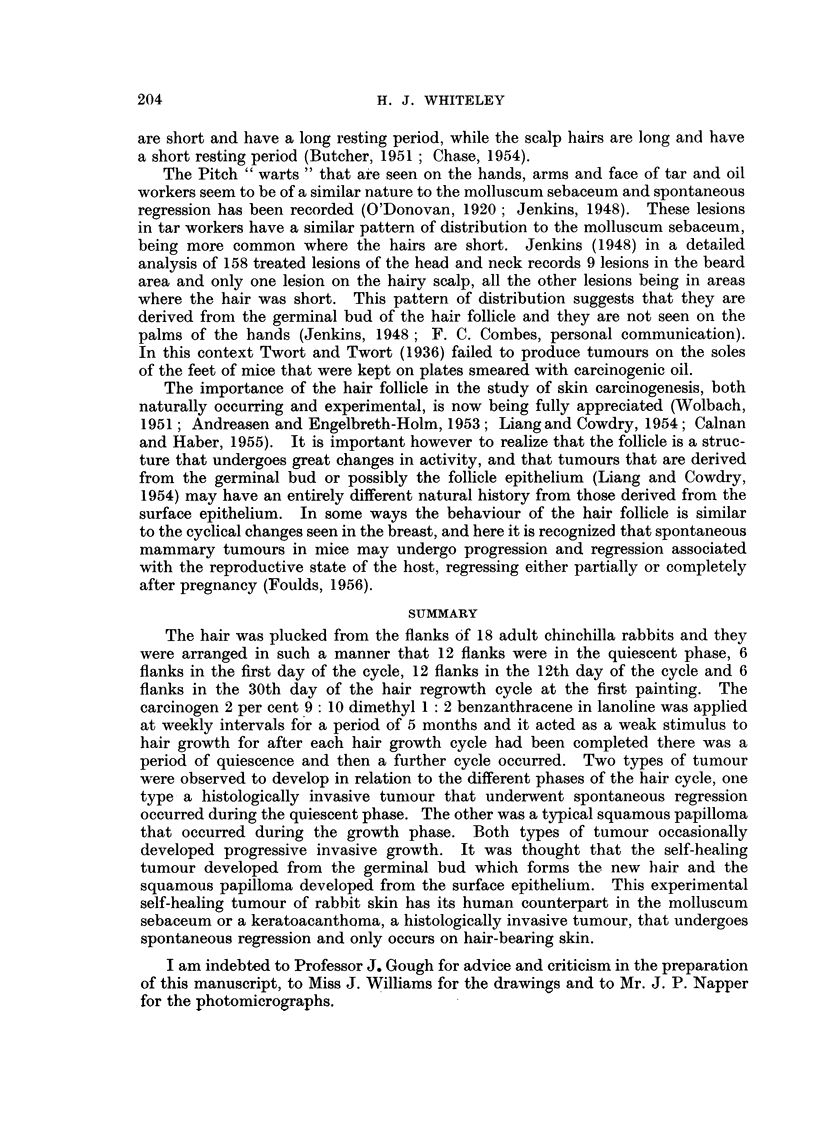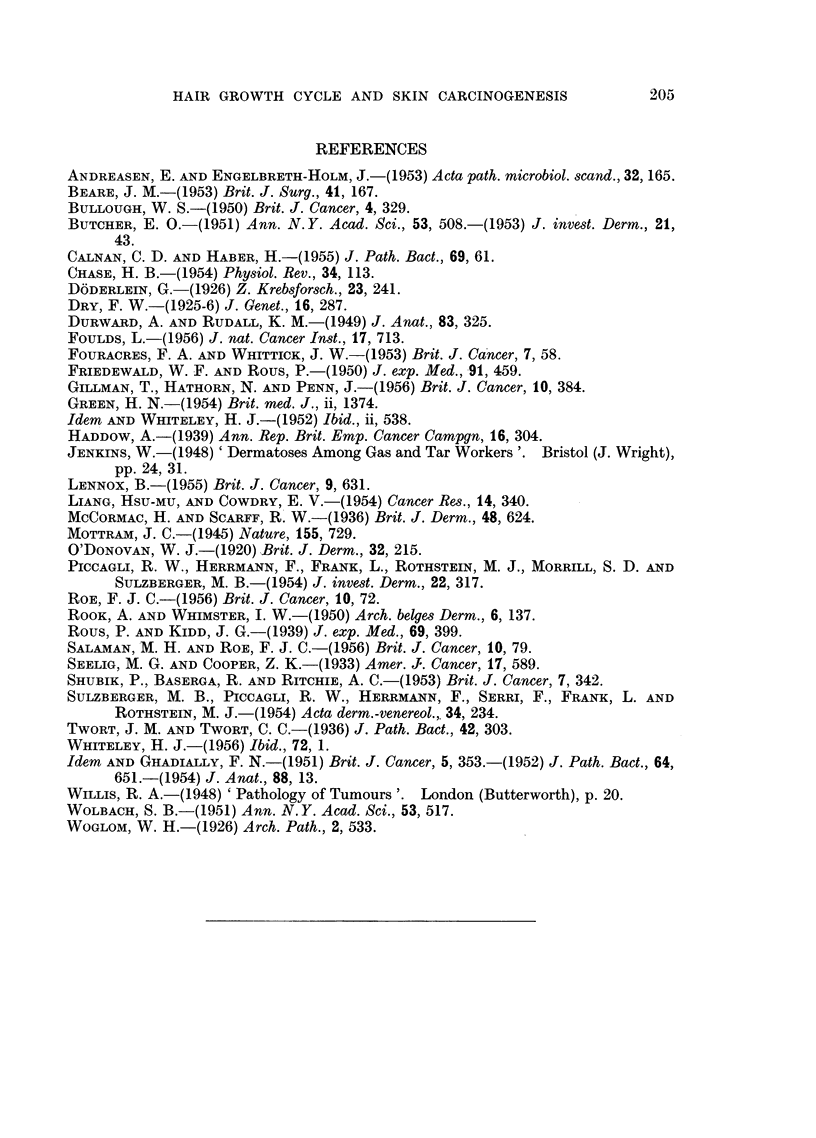# The Effect of the Hair Growth Cycle on Experimental Skin Carcinogenesis in the Rabbit

**DOI:** 10.1038/bjc.1957.27

**Published:** 1957-06

**Authors:** H. J. Whiteley

## Abstract

**Images:**


### THE EFFECT OF THE HAIR GROWTH CYCLE ON EXPERIMENTAL SKIN CARCINOGENESIS IN THE RABBIT

H. J. WHITELEY

*From the Department of Pathology, The Welsh National School of Medicine, Cardiff*

Received for publication March 11, 1957

THE process of hair replacement in the rabbit is discontinuous. It occurs in the form of cyclical waves of active hair growth which spread over the flank from the dorsum to the venter (Whiteley and Ghadially, 1954) and is essentially similar to that occurring in the mouse and rat (Dry, 1925-6 ; Durward and Rudall, 1949). Between the periods of active hair growth, which occur once or twice a year, the skin is in a quiescent state and there is no growth of hair ; during this period the hair growth cycle can be artificially stimulated in any particular part of the skin by plucking out the overlying hair (Whiteley, 1956). It is therefore possible to have known areas of skin in known stages of the hair growth cycle at any particular time, allowing the accurate determination of the effect of the hair growth cycle on experimentally induced lesions.

Naturally occurring bands of hair growth found on the flank of the rabbit during the process of hair replacement were utilized by Whiteley and Ghadially (1951) in an attempt to determine whether this highly active growth cycle had any effect on experimental skin carcinogenesis. It was found, as a result of painting the flanks of rabbits with 2 per cent 9 : 10-dimethyl-1 : 2 benzanthracene in lanoline, that the initial tumours eventually appeared at the site of the originally active zone of growth present on the flank at the first painting.

However, these natural zones of growth were irregular in distribution and there was no uniformity in the stage of the hair growth cycle of individual hairs throughout the zone of growth (Whiteley and Ghadially, 1954). It was therefore decided to amplify these original observations by applying the carcinogen to areas of skin in known stages of the hair growth cycle and to determine more accurately the effect of the hair cycle on experimental carcinogenesis. This paper is the report of the effect of the hair regrowth cycle on experimental skin carcinogenesis. Two types of tumour developed in relation to the different phases of the hair growth cycle, one type a histologically invasive tumour that underwent spontaneous regression occurred during the quiescent phase, and the other a typical squamous papilloma occurred during the growth phase. Both types of tumour occasionally developed progressive invasive growth.

### METHODS

Nine adult male and 9 adult female chinchilla rabbits were used, the animals being housed in individual cages and fed bran mash *ad libitum* with supplements of greens. The hair was plucked manually from both flanks of all the rabbits in such a manner that 12 flanks were in the quiescent phase, 6 flanks in the 1st day of the cycle, 12 flanks in the 12th day of the cycle and 6 flanks in the 30th day of the cycle at the time of the first painting (Fig. 1). The carcinogen, 2 per cent W/W 9 : 10-dimethyl-1 : 2 benzanthracene (DMBA) in lanoline, heated to 40° C. to facilitate application, was painted on the flanks at weekly intervals for 5 months, and the animals were observed for a further 7 months. Before each painting

FIG. 1.—Diagrammatic representation of the stages of the hair growth cycles and the subsequent patterns of hair growth of the flanks in the quiescent phase, 1st day of cycle, 12th day of cycle and 30th day of cycle at first painting (number of flanks in each group in parenthesis). After each cycle was completed there was a period of quiescence and then a further cycle occurred.

the hair was removed with clippers and the animals were photographed. Clipping the hair does not modify the hair growth cycle. Complete or partial biopsies of tumours were taken under ether anaesthesia. These were immediately fixed in alcoholic Bouin and sections were cut at $7\mu$ and stained with haematoxylin and eosin. The skins of the animals that died or were killed during the experiment were stretched out on blotting paper and fixed in formol saline.

### RESULTS

The carcinogen acted as a stimulus to hair growth the cycle starting after a period of 2 to 3 weeks in all the 12 flanks that were in the quiescent phase at first painting. The stimulus was not as strong as that associated with plucking which caused the first signs of hair growth to appear after 1 week. After each hair growth cycle was completed there was a period of quiescence varying from 2 to 8 weeks when a further cycle occurred, so that at any particular time after the first painting there were flanks in all stages of the hair growth cycle (Fig. 1).

It became quite clear that two types of tumour developed and that their appearance was related to the phases of the hair growth cycle (Fig. 2). The squamous papillomata usually occurred during the phase of hair growth, and the self-healing tumour during the quiescent phase. Both these types of tumour occasionally underwent a change to progressive invasive growth.

Attention was directed principally to the self-healing tumour because of its unusual behaviour. Its development and fate will be described first, and then that of the squamous papilloma which apart from its relation to the hair cycle has already been extensively studied in the rabbit (Rous and Kidd 1939).

FIG. 2.—First appearance of the two types of tumour in relation to the stages of the hair growth cycle in four flanks all in different stages of the hair growth cycle at the first painting.

### *The Self-healing Tumour*

Sixteen of the 18 rabbits developed self-healing tumours and the total number and fate of the tumours that developed during the course of the experiment are shown in Table I, the first tumour appearing after 47 days. The percentage of remaining tumours is high as some animals died or were killed while tumours were present, and some animals are still alive.

TABLE I.—*Showing Number and Fate of the Self-healing Tumours that Developed during the Experiment.*

| | |
|---|---|
| Total number of tumours | 296 |
| Number of complete biopsies | 42 |
| Number excluding biopsies | 254 |
| Percentage regression | 75 |
| Percentage undergoing progressive invasive growth | 3 |
| Percentage uncertain behaviour, death during experiment or tumour still present | 22 |

The tumours developed principally during the quiescent phase of the hair cycle but sometimes at the commencement or termination of the period of growth. They did not develop during the period of active hair growth. Occasionally, however, during a period when the painted area was in a growth phase, tumours did develop in the quiescent skin adjacent to the growing edge of the painted area. The occurrence of tumours at the edge of the area of painting has been noted by other observers (Haddow, 1939). The tumours were often multiple, with regressing and developing tumours occasionally occurring on the same flank. Tumours continued to appear after the cessation of painting throughout the duration of the period of observation.

It has been suggested that lanoline is an unsuitable vehicle for the application of a carcinogen as it causes tumour inhibition (Gillman, Hathorn and Penn, 1956). While this may be true, it does not appear to be a factor in the development of the self-healing tumour as these tumours were observed during preliminary studies using oleic acid as a vehicle for the carcinogen DMBA.

*Macroscopical appearance*

The self-healing tumour was quite characteristic, starting as a raised reddened mound covered by normal skin (Fig. 3) it rapidly enlarged up to 2–3 cms. in diameter and the surface became ulcerated (Fig. 4). The degree of ulceration became progressively more pronounced and the lesion occasionally became deeply cratered (Fig. 5). Then there was very rapid resolution of the tumour with healing and the formation of a scar (Fig. 6). Sometimes the bulk of the tumour dropped off leaving a partially epithelialized crater (Fig. 11) which subsequently healed, the whole process taking 3–8 weeks. There was no relationship between the process of regression and the cessation of the hair growth cycle. It was observed, usually towards the end of the period of painting, that sometimes what appeared to be initially a typical self-healing tumour grew progressively and formed a large ulcerated infected lesion with spread under the skin. After the animal had been killed spread to the regional nodes was observed.

On section the early stages were characteristic and quite different from the squamous papilloma. The lesion was spherical in outline lying primarily in the dermis and forming a dome-shaped swelling on the skin. There was often a central cavity containing epithelial debris and this was surrounded by strands of tumour invading the dermis down to the panniculus carnosus (Fig. 7). The surface epithelium over the lesion was thickened and sometimes showed active hair growth and the centre was covered by coagulated exudate. As the lesion developed there was a profound stromal reaction in and around the tumour forming a dense collagenous zone in the dermis (Fig. 8).

*Microscopical appearances of the self-healing tumour*

There was little variation within individual lesions of the cytological characteristics of the tumour cells, which were all of epithelial origin. There was, however, wide variation in appearances in different lesions varying from a spindle cell type showing numerous mitoses and wide infiltration of the dermis to a relatively well-differentiated type showing the formation of epithelial pearls (Fig. 9). The profound stromal reaction that developed was composed of actively proliferating fibroblasts and there was a lymphocytic and large mononuclear cell exudate.

Because it might be argued that complete biopsies from 42 lesions might not be representative of all the tumours, diagnostic biopsies were taken of part of 19 typical lesions all of which ultimately underwent regression and they all showed a similar histological picture of irregular strands of epithelial cells invading the dermis (Fig. 10). In some cases the biopsy operation seemed temporarily to stimulate the lesions to more active growth but regression ultimately took place. Penetration of the panniculus carnosus was never observed in any of the 42 complete biopsies examined.

Complete biopsies were taken from 10 regressing lesions, and there was complete absence of tumour cells in all of the lesions. During the process of regression a crater-like ulcer formed with an overlying mass of keratin and inflammatory exudate (Fig. 11). In the base there was a predominantly lymphocytic and plasma cell infiltration often surrounding completely keratinized epithelial pearls (Fig. 12) some of which were infiltrated with polymorphs and partly surrounded by giant cells (Fig. 13). Serial biopsies from 5 lesions showed that the clumps of tumour cells became vacuolated and infiltrated with polymorphs. There was, in some cases, necrosis of the tumour cells and in others the maturation of the tumour cells into clumps of keratin. Both processes probably occurred to a varying degree in all lesions. When the lesions had healed there was a residual scar in the dermis which was covered by hyperplastic epithelium with associated destruction of the hair follicles in the region of the lesion (Fig. 14).

A few of the lesions that were initially typical self-healing tumours became hyperkeratotic and indistinguishable from the squamous papillomata although they underwent eventual regression. However histological examination revealed that they were not true papilloma as these lesions showed the hyperplastic stromal changes in the base with the other changes associated with a regressing lesion. They were considered to be a variety of self-healing tumour in which the surface epithelium became unduly hyperplastic, thus masking the phase of regression in the deeper parts of the tumour.

*Progressive invasive growth*

Occasionally towards the end of the period of painting it was noticed that what appeared to be a typical self-healing tumour did not undergo regression but grew progressively becoming deeply ulcerated and infected. There was an invasive

---

EXPLANATION OF PLATES.

FIG. 3.—Typical self-healing tumour which had been present for 7 days. The tumour developed during a quiescent phase 110 days after the first paiting. × 1

FIG. 4.—After a further 7 days, the tumour is now 2·5 cm. diameter. × 1

FIG. 5.—After a further 7 days. The tumour is now deeply cratered and has raised edges. × 1

FIG. 6.—After a further 7 days the tumour had completely disappeared leaving a scar. × 1

FIG. 7.—Complete biopsy of typical self-healing tumour that had been present at 7 days. There is invasion down to the panniculus carnosus. H. & E. × $4\frac{4}{5}$.

FIG. 8.—Complete biopsy from a self-healing tumour that had been present for 14 days. There is a considerable degree of stromal reaction, particularly in the deepest part of the lesion. H. & E. × $4\frac{4}{5}$.

FIG. 9.—High power from Fig. 7 showing the deepest part of the edge of the lesion which is composed of infiltrating groups of epithelial cells, some forming epithelial pearls. H. & E. × 57.

FIG. 10.—Partial biopsy taken from a typical self-healing tumour that developed in a quiescent zone 112 days after first painting. The features are those of an invasive squamous cell carcinoma, but the tumour underwent complete regression. H. & E. × 60.

FIG. 11.—Complete biopsy of a typical self-healing tumour that had been present for 4 weeks and was undergoing regression. There had been complete disappearance of all the tumour cells. H. & E × $4\frac{4}{5}$.

FIG. 12.—Self-healing tumour undergoing regression. There are large completely keratinized epithelial pearls in the dermis surrounded by a giant cell and lymphocytic cell infiltrate. H. & E. × 47.

FIG. 13.—High power from Fig. 11 showing the infiltration of the epithelial pearls with polymorphs and the surrounding giant cell reaction. H. & E. × 69.

FIG. 14.—Complete biopsy from a self-healing tumour that had undergone complete regression. There is a scar in the dermis with overlying hyperplastic epithelium, but there has been no regeneration of the hair follicles. H. & E. × $4\frac{4}{5}$.

FIG. 15.—Rabbit flank in the 3rd week of the second hair growth cycle, showing the appearance of multiple small papillomata. There were no tumours present during the preceding quiescent phase.

FIG. 16.—Complete biopsy of a typical squamous papilloma that developed during a growth cycle 63 days after the first painting and which had been present for 7 weeks. The appearance is quite different from the self-healing tumour. H. & E. × $5\frac{3}{4}$.

FIG. 17.—Showing the persistence of active hair follicles between areas of hyperplastic epithelium of an early squamous papilloma. H. & E. × 36.

3

4

5

6

Whiteley.

7

8

11

14

9

10

Whiteley.

12

13

15

16

17

Whiteley.

growth under the skin and on section it was seen that the tumour had penetrated the panniculus carnosus. Of the self-healing tumours 3 per cent underwent this change to progressive invasive growth. The cytological appearances of the tumour cells, apart from a slight increase in pleomorphism, were very similar to those of the self-healing tumour, and the appearance of the metastases in the lymph nodes were similar to the primary lesion. It was therefore impossible to correlate the behaviour of a tumour with its cytological characteristics.

### *Squamous Papillomata*

The development of the squamous papillomata was clearly related to the cycle as tumours developed principally during the phase of hair growth (Fig. 15) as many as 20 tumours occurring simultaneously on one flank. In all 598 tumours were observed and their appearance was characteristic and quite different from that of the self-healing tumour (Fig. 16) in that they appeared to arise from the surface epithelium and grow outward. In some of the early tumours examined during the phase of hair growth, normal active hair follicles were seen growing between areas of the hyperplastic epithelium of the squamous papilloma (Fig. 17). The behaviour and fate of these tumours has been carefully described by Rous and Kidd (1939) and the findings in this experiment are in agreement with their observations on the behaviour of the "common papillomata". These tumours seem to be dependent on the stimulus of the carcinogen for their survival as there was gradual regression of the lesions after the cessation of painting (Rous and Kidd, 1939). Regression did not appear to be related to the subsequent development of a phase of hair growth as was described in the mouse by Mottram (1945).

The change to progressive invasive growth with invasion of the panniculus carnosus was observed in 11 of the squamous papillomata although the lesions did not become deeply ulcerated as did those of the self-healing carcinoma.

### *Tumour Transplantation and Tissue Culture*

Attempts were made to transplant some of the self-healing tumours into other sites in the same animal, and into other animals, some under the influence of cortisone, as cortisone has been shown to facilitate the take of tumour transplants (Green and Whiteley, 1952), but in no case could a successful result be obtained. With the co-operation of Dr. A. D. Evans attempts were made to see whether the cells of the self-healing tumour would proliferate or undergo spontaneous regression in tissue cultures, but results so far have been inconclusive. Both these lines of investigation are being pursued to determine whether the spontaneous regression is an intrinsic property of the tumour cells or is the result of local or general resistance to the tumour.

### DISCUSSION

The early workers on tar cancer, whose findings are extensively reviewed by Woglom (1926), Seelig and Cooper (1933) and Rous and Kidd (1939), described in the rabbit a type of tumour having the histological features of a squamous cell carcinoma but which underwent spontaneous regression. This type of tumour was variously called a cancroid, a carcinoid, or a carcinomatoid as it was thought to represent a stage in the development of a true invasive neoplasm and that it was dependent on the stimulus of tarring for its survival. As a result of this apparent discrepancy between cytological appearance and behaviour there was considerable discussion as to what features indicated a progressively growing carcinoma, or even whether spontaneous regression should be taken to indicate that the lesion was non malignant. Döderlein (1926) believed that the only true indication of malignancy was invasion of the panniculus carnosus, and this criterion has been adopted by other writers in particular Shubik, Baserga and Ritchie (1953) and Roe (1956).

The self-healing tumours described in this paper are no doubt similar in nature to the carcinomatoids observed on the ear of the rabbit by Rous and Kidd (1939). They used the ear in preference to the flank as the hair is much shorter and finer, in particular on the inner surface. However when the flank was used in the present experiments and the carcinogen applied in a bland solvent, it became apparent that the development of tumours was closely related to the hair growth cycle. The self-healing tumour occurred during the quiescent phase, and the squamous papilloma usually during the growth phase. This relationship became apparent because of the relatively long period of hair growth in the rabbit (7 weeks) and the occurrence of a period of quiescence between the periods of growth.

Profound changes occur in mitotic activity during the hair growth cycle, both in the epidermis and in the hair follicles. The epidermis shows a peak of activity in the early stages of the cycle, and at the same time the quiescent buds from which the new hairs develop are stimulated into intense activity and extend deeply into the dermis, (Whiteley, 1956). There are therefore two proliferating epithelial elements in the skin and these react quite differently in the sense that the epidermis grows outwards and the hair follicles grow into the dermis, this is reflected in their response to the carcinogen. It is generally believed that a benign tumour retains some of the properties of the parent tissue (Willis, 1948). These two types of tumour, which occur at about the same time after the first painting, could represent benign neoplasms of these two different types of epithelium, the self-healing tumour developing from the germinal buds from which the new hairs develop. During the quiescent phase these buds are close to the surface and would be within reach of the carcinogen (Butcher, 1953). This would not be the case during active hair growth when the actively dividing tissue is deep in the dermis. The behaviour of this tumour is closely parallel to that of the hair follicle, rapid growth starting suddenly in quiescent skin, invasion down to the panniculus carnosus, but without invasion of the muscle, and then regression. Tumours would not be expected to occur during the period of growth, as the germinal buds from which tumours might have originated have developed into normal active hair follicles.

The squamous papilloma represents a benign tumour of the surface epithelium which grows outwards as does this epithelium. The appearance of papillomata during the period of hair growth could be related to the rapid peak of mitotic activity occurring after the hair cycle has been initiated, as the yield of tumours from an area treated with carcinogen is higher when the surface is subsequently treated with a mitotic stimulant such as croton oil (Bullough, 1950).

The occurrence of these two types of tumour, one from the germinal bud and the other from the surface epithelium, means that the original hypothesis of Whiteley and Ghadially (1951 and 1952) that all tumours were derived from the follicles is now no longer tenable.

In this investigation the feature of interest is the sudden regression of the self-healing tumours. It has been assumed that the carcinomatoids were dependent on the stimulus of tarring for survival (Rous and Kidd, 1939) but further investigation by Friedewald and Rous (1950) showed that carcinomatoids may develop on rabbits. ears several years after cessation of painting and usually occur at the site of healing punch biopsy holes. The results presented here are in agreement in that the self-healing tumours develop and regress during and after application of carcinogen. It does not seem probable that the regression is due to an immunity reaction similar to that postulated by Green (1954) to account for the regression of homologous tumour transplants, as regressing and developing tumours were found in the same animal, and the period of survival of these tumours appeared to increase during the course of the experiment. This hypothesis would only be possible if each tumour was antigenically distinct or that the immune response was purely local with no systemic counterpart.

The mechanism that controls the duration of growth of hair is not fully understood, nor is the exact sequence of events known when the actively growing follicle changes into the resting state. However it is thought that the bulk of the epithelial cells degenerate but that some form a keratin mass which acts as an anchoring club for the newly formed hair (Chase, 1954). The histological features observed during regression of the tumour are similar as there is destruction of most of the tumour cells with the maturation of some to epithelial pearls. Is the regression due to the fact that the tumour cells are still under the same influences as those that cause the natural regression of the follicle at the end of its growth period?

This type of tumour has not been observed as self healing in the mouse or the rat, but very rapidly growing tumours having initially a similar gross and microscopical appearance have been described in the mouse, the " Carcinoma d'emblée " of Piccagli *et al.* (1954) and Sulzberger *et al.* (1954) and likewise some of the tumours described by Roe (1956) and Salaman and Roe (1956) as probably malignant. In the rat the squamous carcinoma " ex cyst " described by Lennox (1955) is similar. All these tumours possibly arise not from the surface epithelium but from the germinal bud.

This experimentally induced self-healing tumour appears to have a close similarity to the molluscum sebaceum or keratoacanthoma described in man (McCormac and Scarff, 1936; Rook and Whimster, 1950; Fouracres and Whittick, 1953; Beare, 1953) which appears only to occur on hair-bearing skin and is thought to develop from hair follicles (Calnan and Haber, 1955). If the molluscum sebaceum were to develop from the germinal bud instead of the fully developed follicle, as postulated for the self-healing tumour of rabbit skin, it would be expected to occur more frequently in areas of the body where the follicle resting phase is long, for the germinal bud would then have a greater length of exposure to the carcinogenic agents that are thought to be partly responsible for its development. The lesions of molluscum sebaceum in man have in fact a distribution related to the period of hair growth and are found most frequently on the face and ears and to a lesser extent on the scalp. Beare (1953) in a study of 76 cases described 64 lesions occurring on the face, of these 17 were on the nose and 28 on the cheeks, but the exact site of the latter was not specified. He found 10 tumours on the ears and back of the neck, 2 on the forearms, but none were found on the scalp. The hairs on the face and ears, except for the beard area, are short and have a long resting period, while the scalp hairs are long and have a short resting period (Butcher, 1951 ; Chase, 1954).

The Pitch " warts " that are seen on the hands, arms and face of tar and oil workers seem to be of a similar nature to the molluscum sebaceum and spontaneous regression has been recorded (O'Donovan, 1920 ; Jenkins, 1948). These lesions in tar workers have a similar pattern of distribution to the molluscum sebaceum, being more common where the hairs are short. Jenkins (1948) in a detailed analysis of 158 treated lesions of the head and neck records 9 lesions in the beard area and only one lesion on the hairy scalp, all the other lesions being in areas where the hair was short. This pattern of distribution suggests that they are derived from the germinal bud of the hair follicle and they are not seen on the palms of the hands (Jenkins, 1948 ; F. C. Combes, personal communication). In this context Twort and Twort (1936) failed to produce tumours on the soles of the feet of mice that were kept on plates smeared with carcinogenic oil.

The importance of the hair follicle in the study of skin carcinogenesis, both naturally occurring and experimental, is now being fully appreciated (Wolbach, 1951 ; Andreasen and Engelbreth-Holm, 1953 ; Liang and Cowdry, 1954 ; Calnan and Haber, 1955). It is important however to realize that the follicle is a structure that undergoes great changes in activity, and that tumours that are derived from the germinal bud or possibly the follicle epithelium (Liang and Cowdry, 1954) may have an entirely different natural history from those derived from the surface epithelium. In some ways the behaviour of the hair follicle is similar to the cyclical changes seen in the breast, and here it is recognized that spontaneous mammary tumours in mice may undergo progression and regression associated with the reproductive state of the host, regressing either partially or completely after pregnancy (Foulds, 1956).

### SUMMARY

The hair was plucked from the flanks of 18 adult chinchilla rabbits and they were arranged in such a manner that 12 flanks were in the quiescent phase, 6 flanks in the first day of the cycle, 12 flanks in the 12th day of the cycle and 6 flanks in the 30th day of the hair regrowth cycle at the first painting. The carcinogen 2 per cent 9 : 10 dimethyl 1 : 2 benzanthracene in lanoline was applied at weekly intervals for a period of 5 months and it acted as a weak stimulus to hair growth for after each hair growth cycle had been completed there was a period of quiescence and then a further cycle occurred. Two types of tumour were observed to develop in relation to the different phases of the hair cycle, one type a histologically invasive tumour that underwent spontaneous regression occurred during the quiescent phase. The other was a typical squamous papilloma that occurred during the growth phase. Both types of tumour occasionally developed progressive invasive growth. It was thought that the self-healing tumour developed from the germinal bud which forms the new hair and the squamous papilloma developed from the surface epithelium. This experimental self-healing tumour of rabbit skin has its human counterpart in the molluscum sebaceum or a keratoacanthoma, a histologically invasive tumour, that undergoes spontaneous regression and only occurs on hair-bearing skin.

I am indebted to Professor J. Gough for advice and criticism in the preparation of this manuscript, to Miss J. Williams for the drawings and to Mr. J. P. Napper for the photomicrographs.

### REFERENCES

ANDREASEN, E. AND ENGELBRETH-HOLM, J.—(1953) *Acta path. microbiol. scand.*, **32**, 165.
BEARE, J. M.—(1953) *Brit. J. Surg.*, **41**, 167.
BULLOUGH, W. S.—(1950) *Brit. J. Cancer*, **4**, 329.
BUTCHER, E. O.—(1951) *Ann. N.Y. Acad. Sci.*, **53**, 508.—(1953) *J. invest. Derm.*, **21**, 43.
CALNAN, C. D. AND HABER, H.—(1955) *J. Path. Bact.*, **69**, 61.
CHASE, H. B.—(1954) *Physiol. Rev.*, **34**, 113.
DÖDERLEIN, G.—(1926) *Z. Krebsforsch.*, **23**, 241.
DRY, F. W.—(1925-6) *J. Genet.*, **16**, 287.
DURWARD, A. AND RUDALL, K. M.—(1949) *J. Anat.*, **83**, 325.
FOULDS, L.—(1956) *J. nat. Cancer Inst.*, **17**, 713.
FOURACRES, F. A. AND WHITTICK, J. W.—(1953) *Brit. J. Cancer*, **7**, 58.
FRIEDEWALD, W. F. AND ROUS, P.—(1950) *J. exp. Med.*, **91**, 459.
GILLMAN, T., HATHORN, N. AND PENN, J.—(1956) *Brit. J. Cancer*, **10**, 384.
GREEN, H. N.—(1954) *Brit. med. J.*, ii, 1374.
*Idem* AND WHITELEY, H. J.—(1952) *Ibid.*, ii, 538.
HADDOW, A.—(1939) *Ann. Rep. Brit. Emp. Cancer Campgn*, **16**, 304.
JENKINS, W.—(1948) ' Dermatoses Among Gas and Tar Workers '. Bristol (J. Wright), pp. 24, 31.
LENNOX, B.—(1955) *Brit. J. Cancer*, **9**, 631.
LIANG, HSU-MU, AND COWDRY, E. V.—(1954) *Cancer Res.*, **14**, 340.
McCORMAC, H. AND SCARFF, R. W.—(1936) *Brit. J. Derm.*, **48**, 624.
MOTTRAM, J. C.—(1945) *Nature*, **155**, 729.
O'DONOVAN, W. J.—(1920) *Brit. J. Derm.*, **32**, 215.
PICCAGLI, R. W., HERRMANN, F., FRANK, L., ROTHSTEIN, M. J., MORRILL, S. D. AND SULZBERGER, M. B.—(1954) *J. invest. Derm.*, **22**, 317.
ROE, F. J. C.—(1956) *Brit. J. Cancer*, **10**, 72.
ROOK, A. AND WHIMSTER, I. W.—(1950) *Arch. belges Derm.*, **6**, 137.
ROUS, P. AND KIDD, J. G.—(1939) *J. exp. Med.*, **69**, 399.
SALAMAN, M. H. AND ROE, F. J. C.—(1956) *Brit. J. Cancer*, **10**, 79.
SEELIG, M. G. AND COOPER, Z. K.—(1933) *Amer. J. Cancer*, **17**, 589.
SHUBIK, P., BASERGA, R. AND RITCHIE, A. C.—(1953) *Brit. J. Cancer*, **7**, 342.
SULZBERGER, M. B., PICCAGLI, R. W., HERRMANN, F., SERRI, F., FRANK, L. AND ROTHSTEIN, M. J.—(1954) *Acta derm.-venereol.*, **34**, 234.
TWORT, J. M. AND TWORT, C. C.—(1936) *J. Path. Bact.*, **42**, 303.
WHITELEY, H. J.—(1956) *Ibid.*, **72**, 1.
*Idem* AND GHADIALLY, F. N.—(1951) *Brit. J. Cancer*, **5**, 353.—(1952) *J. Path. Bact.*, **64**, 651.—(1954) *J. Anat.*, **88**, 13.
WILLIS, R. A.—(1948) ' Pathology of Tumours '. London (Butterworth), p. 20.
WOLBACH, S. B.—(1951) *Ann. N.Y. Acad. Sci.*, **53**, 517.
WOGLOM, W. H.—(1926) *Arch. Path.*, **2**, 533.